# Vaginal Biomarkers That Predict Cervical Length and Dominant Bacteria in the Vaginal Microbiomes of Pregnant Women

**DOI:** 10.1128/mBio.02242-19

**Published:** 2019-10-22

**Authors:** Steven S. Witkin, Antonio F. Moron, Benjamin J. Ridenhour, Evelyn Minis, Alan Hatanaka, Stephanno G. P. Sarmento, Marcelo S. Franca, Francisco H. C. Carvalho, Tatiana K. Hamamoto, Rosiane Mattar, Ester Sabino, Iara M. Linhares, Marilza V. C. Rudge, Larry J. Forney

**Affiliations:** a Department of Obstetrics and Gynecology, Weill Cornell Medicine, New York, New York, USA; b Institute of Tropical Medicine, University of São Paulo, São Paulo, Brazil; c Department of Obstetrics, Federal University of Sao Paulo, Sao Paulo, Brazil; d Department of Mathematics, University of Idaho, Moscow, Idaho, USA; e Center for Modeling Complex Interactions, University of Idaho, Moscow, Idaho, USA; f Department of Biological Sciences, University of Idaho, Moscow, Idaho, USA; g Institute for Bioinformatics and Evolutionary Studies, University of Idaho, Moscow, Idaho, USA; h Department of Gynecology and Obstetrics, Federal University of Ceara, Ceara, Brazil; i Department of Obstetrics and Gynecology, Sao Paulo University Medical School, Sao Paulo, Brazil; j Department of Obstetrics and Gynecology, Sao Paulo State University Medical School, Botucatu, Brazil

**Keywords:** cervical length, d-lactic acid, preterm birth, TIMP-1, vaginal microbiome, *Lactobacillus*, cervix, lactic acid, microbial communities

## Abstract

In many impoverished regions of the world, it may not be possible to assess two major risk factors for preterm birth: a short cervical length and the depletion of vaginal lactobacilli. We determined whether measuring specific compounds in vaginal fluid might be a simple, noninvasive, and cost-effective way to predict the bacteria that dominate the vaginal microbiome and indicate the presence of a shortened cervix (<25 mm). Vaginal fluid samples were prospectively collected from mid-trimester pregnant women, and the concentrations of d- and l-lactic acid, tissue inhibitor of matrix metalloproteinases TIMP-1 and TIMP-2, matrix metalloproteinases MMP-2 and MMP-8, the 70-kDa heat shock protein, a2 isoform of vacuolar ATPase, and sequestrome-1 were quantified by an enzyme-linked immunosorbent assay (ELISA). The compositions of vaginal microbiomes were assessed by analysis of the V1-V3 regions of 16S rRNA genes, while cervical length was determined by transvaginal ultrasonography. The vaginal microbiomes could be clustered into five community state types (CSTs), four of which were dominated by a single *Lactobacillus* species. The dominance of Lactobacillus crispatus or Lactobacillus jensenii in the vaginal microbiome predicted the level of d-lactic acid present. Several of the biomarkers, especially TIMP-1, in combination with the subject’s age and race, were significantly associated with cervical length. Using piecewise structural equation modeling, we established a causal network that links CST to cervical length via biomarkers. We concluded that measuring levels of TIMP-1 and d-lactic acid in vaginal secretions might be a straightforward way to assess the risk for preterm birth due to a short cervix and microbiome composition.

## Abstract (precis)

Premature birth and its complications are the largest contributors to infant death in the United States and globally. A short cervical length and the depletion of *Lactobacillus* species are known risk factors for preterm birth. However, in many resource-poor areas of the world, the technology to test for their occurrence is unavailable, and pregnant women with these risk factors are neither identified nor treated. In this study, we used path analysis to gain an unprecedented understanding of interactions between vaginal microbiome composition, the concentrations of various compounds in vaginal secretions, and cervical length. We identified low-cost point-of-care measures that might be used to identify pregnant women at risk for preterm birth. The use of these measures coupled with appropriate preventative or treatment strategies could reduce the incidence of preterm births in poor areas of the world that lack access to more sophisticated diagnostic methods.

## INTRODUCTION

Complications of preterm birth are the single largest cause of neonatal deaths and account for 35% of the world’s 3.1 million neonatal deaths each year (1, 2). Two factors have consistently been associated with an increased occurrence of preterm birth. One is the presence of a short cervix, (typically defined as <25 mm) (3, 4). More recent investigations have highlighted the influence of the composition of the vaginal microbiome on cervical length during pregnancy (5, 6) and different susceptibilities to adverse outcomes when Lactobacillus crispatus is absent or present only at a low level in the vaginal microbiome and displaced by Lactobacillus iners, Gardnerella vaginalis, or other bacterial species (5–10). In prosperous locations, most pregnant women are routinely examined to determine cervical length by vaginal ultrasound at 18 to 24 weeks gestation (11), and if they are found to have a short cervix, they are treated with progesterone, a cervical cerclage, or a cervical pessary to reduce the likelihood of premature delivery (12). Similarly, if pregnant women show signs of bacterial vaginosis by microscopy of Gram-stained smears or various diagnostics based on gene amplification, they may be offered treatment with antibiotics in an attempt to restore the dominance of lactobacilli (13). The resources needed to perform a vaginal ultrasound or characterize the composition of vaginal bacterial communities are often not available in many areas of the world. Consequently, the rates of preterm birth and its deleterious consequences are more prevalent in these regions (14). There remains an unmet need to develop an inexpensive point-of-care method to identify pregnant women in areas with limited resources who may be at increased risk for preterm birth due to a short cervix or the predominance of bacteria other than lactobacilli.

Lactic acid is the principal acid in vaginal secretions, and it is responsible for acidification of the vagina. The majority of vaginal lactic acid results from the fermentation of glycogen breakdown products by four species of lactobacilli, namely, Lactobacillus crispatus, L. iners, L. jensenii, and L. gasseri. Vaginal epithelial cells also produce and release a small quantity of lactic acid (15). The vaginal epithelial cells as well as *L. iners* produce only the l-lactic acid isomer, while. L. jensenii produces only the d-isomer, and L. crispatus and L. gasseri produce both d- and l-lactic acid (16). Thus, the level of d-lactic acid in the vagina may indicate which bacterial species are dominant.

The findings of previous studies suggest that other biomarkers may be associated with differences in cervical length. These biomarkers include tissue inhibitor of metalloproteinases TIMP-1 and TIMP-2, matrix metalloproteinases MMP-2 and MMP-8, p62 (also known as sequestosome-1), the a2 isoform of vacuolar ATPase (a2V), and the inducible heat shock protein Hsp70. The relative concentrations of TIMPs and MMPs have been shown to influence the ability of bacteria to affect properties of the uterine cervix (17), while p62 is an intracellular protein that marks degraded intracellular macromolecules and microorganisms for destruction by autophagy (18). p62 is consumed during this process, and so the intracellular level of p62 is inversely related to the extent of autophagy. a2V is thought to regulate the immune response during pregnancy and may play a role in infection-induced preterm birth. For example, suppression of a2V expression in mice induces preterm labor (19). Finally, intra-amniotic infections have been associated with higher levels of Hsp70 (20), and in turn, Hsp70 upregulation has been linked to preterm delivery (21), suggesting a pathway for bacterial infections to induce preterm birth.

In the present study, we used path analysis to evaluate interactions between vaginal microbiome composition, the concentrations of various compounds in vaginal secretions, and cervical length. Our aim was to identify low-cost point-of-care measures that might be used to identify pregnant women with a shortened cervix or an altered vaginal microbiome.

## RESULTS

The characteristics of the study population of 340 women used for the analyses of cervical length and vaginal compound covariates are shown in Table 1. The mean age of subjects was 29.1 years, the mean body mass index was 27.5 kg/m^2^, the mean cervical length was 32.9 mm, and 10.6% of the women had a short cervix. The mean gestational age at the time of sample collection was 21.5 weeks, and the gestational age at the time of delivery was 38.2 weeks; 16.5% of subjects had a preterm birth (delivery at <37 weeks gestation). A larger set of samples (*n* = 629) was used to characterize the species composition of vaginal microbiomes (see Table S1 in the supplemental material).

**TABLE 1:** Demographic data on 340 study subjects

Characteristic	No. of women or % of women	Value for characteristic		
		Mean	SD^*a*^	Median
Age (yr)	340	29.1	7.3	29.0
Body mass index (kg/m^2^)	340	27.5	6.4	26.3
Race	340			
White (%)	53.2%			
Mixed (%)	36.8%			
Black (%)	10.0%			
Cervical length (mm)	340	32.9	8.5	33.4
Short cervix (<25 mm)	340	10.6%		
Gravidity	340	2.4	1.6	2.0
Parity	340	1.0	1.1	1.0
Gestational age sample (wk)	340	21.5	1.4	21.4
Gestational age delivery (wk)	261	38.2	2.7	38.9
Preterm birth (<37 wk)	261	16.5%		

a SD, standard deviation.

10.1128/mBio.02242-19.1 TABLE S1 Demographic data on 629 women used for microbiome analysis. Download Table S1, DOCX file, 0.01 MB. Copyright © 2019 Witkin et al. 2019 Witkin et al. This content is distributed under the terms of the Creative Commons Attribution 4.0 International license.

### Vaginal microbiomes of study subjects.

**FIG 1:**
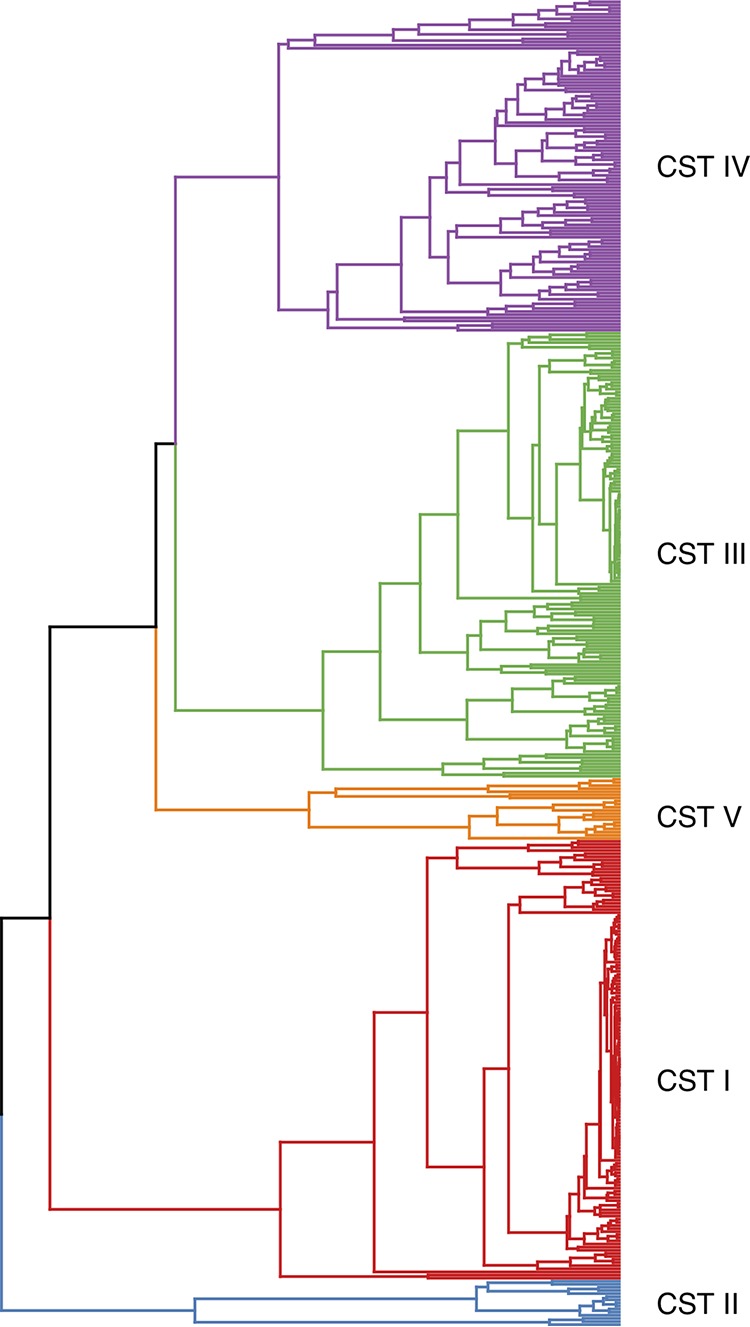
Dendrogram of the 428 uniquely observed microbiomes in this study. The dendrogram was created using the methods of Anderson et al. (44) to calculate the distance between communities. UPGMA was utilized to create the dendrogram, and the number of clusters was determined via silhouette analysis. The different CSTs, which correspond to the five identified clusters, are highlighted.

**FIG 2:**
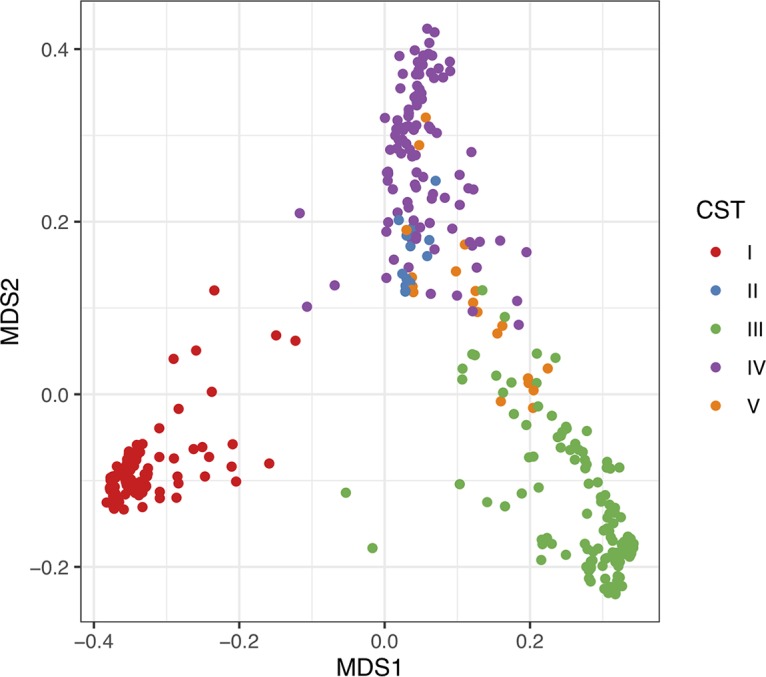
Plot of communities in two-dimensional space after transformation via NMDS into a five-dimensional space. Distances between communities were calculated as described by Anderson et al. (44) prior to transformation. The stress score of the resulting NMDS was quite low (0.066). The first two axes of the transformed data are plotted.

The microbiome compositions of the samples were very similar to those observed in other studies of the vaginal microbiome (Table S2) (22, 23). L. crispatus was by far the most dominant species observed, followed by *L. iners* and G. vaginalis. The vaginal community compositions of 204 individuals were highly skewed and contained >99.9% of single species. Of these, 122, 63, 11, 6, and 2 were dominated by L. crispatus, *L. iners*, *G. vaginalis*, L. jensenii, and L. gasseri, respectively. In contrast, there were three subjects within community state type (CST) IV with communities that exhibited high evenness, and these were comprised of taxa that are normally uncommon in the vaginal microbiome. After collapsing identical microbiomes, there were 428 unique microbiome compositions observed. Using silhouette analyses, these unique microbiomes clearly resolved into five CSTs (Fig. 1). The average composition of each CST along with the number of women within each CST cluster are shown in Table 2. CST I was dominated by L. crispatus, CST II was dominated by L. gasseri, CST III was dominated by *L. iners*, CST IV exhibited greater evenness but was dominated by *G. vaginalis*, and CST V was dominated by L. jensenii. Rescaling the communities to a five-dimensional space using nonmetric dimensional scaling (NMDS) produced a very low stress value with only ∼6.6% information loss, and a plot of the communities according to the first two axes of the NMDS ordination is shown in Fig. 2.

**TABLE 2:** Average relative abundance of the five community state types and the entire study group

Bacterium	Community state type					
	I (*N* = 263^*a*^)	II (*N* = 16)	III (*N* = 206)	IV (*N* = 119)	V (*N* = 25)	All (*N* = 629)
L. crispatus	0.964	0.002	0.020	0.016	0.008	0.414
L. gasseri	0.001	0.856	0.003	0.031	0.000	0.023
*L. iners*	0.015	0.001	0.871	0.075	0.199	0.296
*G. vaginalis*	0.005	0.037	0.026	0.575	0.028	0.143
L. jensenii	0.008	0.000	0.045	0.011	0.751	0.055
*A. vaginae*^*b*^	0.001	0.001	0.004	0.049	0.000	0.012
*Megasphaera*	0.000	0.000	0.008	0.034	0.000	0.005
*A. christensensii*^*c*^	0.000	0.000	0.001	0.007	0.000	0.003
*Prevotella*	0.000	0.000	0.001	0.008	0.000	0.001
Other^*d*^	0.007	0.104	0.021	0.194	0.012	0.049

a *N* is the number of individuals in each group.

b *Atopobium vaginae*.

c *Aerococcus christensensii*.

d The sum of all other taxa in the communities of each CST.

10.1128/mBio.02242-19.2 TABLE S2 Composition of vaginal communities. Download Table S2, XLSX file, 0.4 MB. Copyright © 2019 Witkin et al. 2019 Witkin et al. This content is distributed under the terms of the Creative Commons Attribution 4.0 International license.

### Association of vaginal community state types and components of vaginal secretions.

**FIG 3:**
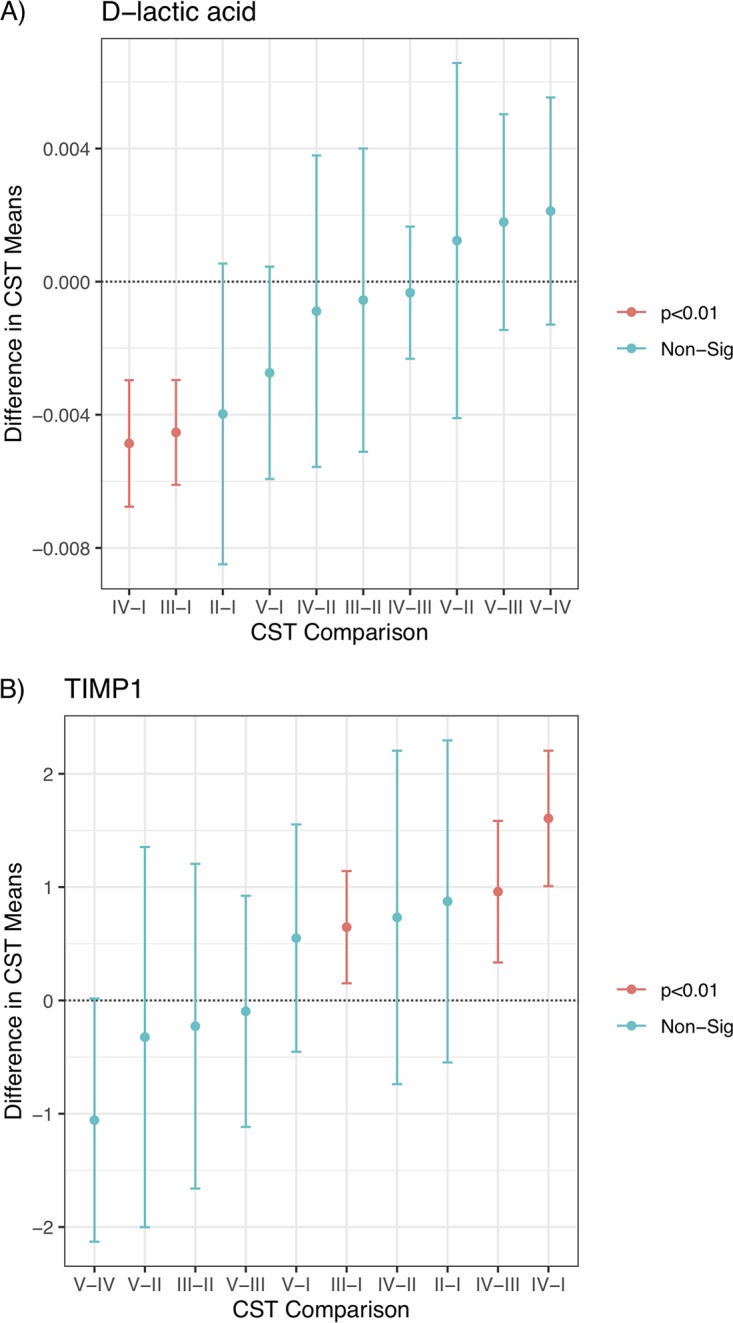
Plot of the pairwise differences in CST means for d-lactic acid (A) and TIMP-1 (B). The *x* axis indicates which CSTs were compared, and the arithmetic relationships were plotted. The central point is the estimate, and the bars indicate the 95% confidence interval of the difference; significance is given by the color of the bar and calculated using a Tukey test. Non-Sig, nonsignificant.

After determining the CST associated with each individual woman, we tested whether either cervical length or the concentrations of vaginal compounds were associated with a particular CST. Four of the compounds showed no association with CST (MMP-2, Hsp70, a2V, and p62), while five compounds showed significant associations. These compounds included d-lactic acid (*F*_4,335_ = 20.776, *P* < 0.001), l-lactic acid (*F*_4,335_ = 3.52, *P* = 0.008), TIMP-1 (*F*_4,335_ = 13.938, *P* < 0.001), TIMP-2 (*F*_4,335_ = 5.793, *P* < 0.001), and MMP-8 (*F*_4,335_ = 2.543, *P* = 0.040). *Post hoc* Tukey tests showed which CSTs differed from the others. In general, comparisons with CST II and CST V were nonsignificant, most likely due to the small sample sizes for these groups (16 and 25, respectively). The levels of d-lactic acid were highest in communities of CST I, followed by communities of CST V (Fig. 3A). High levels of l-lactic acid were associated with CST III (see Fig. S1 in the supplemental material). TIMP-1 was most positively associated with communities of CST IV and CST III, while CST I had the least TIMP-1 (Fig. 3B). TIMP-2 and MMP-8 exhibited a pattern that was very similar to TIMP-1 (Fig. S2 and S3). The correlations between these variables were 0.66 (TIMP-1−TIMP-2), 0.52 (TIMP-1−MMP-8), and 0.59 (TIMP-2−MMP-8), confirming the similarity observed in the Tukey tests (Fig. S4).

10.1128/mBio.02242-19.3 FIG S1 Plot of the pairwise differences in CST means for l-lactic acid. The *x* axis indicates which CSTs were compared, and the arithmetic relationship was plotted. The central point is the estimate, and the bars indicate the 95% confidence interval on the difference; significance is given by the color of the bar and calculated using a Tukey test. Download FIG S1, DOCX file, 0.03 MB. Copyright © 2019 Witkin et al. 2019 Witkin et al. This content is distributed under the terms of the Creative Commons Attribution 4.0 International license.

10.1128/mBio.02242-19.4 FIG S2 Plot of the pairwise differences in CST means for TIMP-2. The *x* axis indicates which CSTs were compared, and the arithmetic relationship was plotted. The central point is the estimate, and the bars indicate the 95% confidence interval on the difference; significance is given by the color of the bar and calculated using a Tukey test. Download FIG S2, DOCX file, 0.03 MB. Copyright © 2019 Witkin et al. 2019 Witkin et al. This content is distributed under the terms of the Creative Commons Attribution 4.0 International license.

10.1128/mBio.02242-19.5 FIG S3 Plot of the pairwise differences in CST means for MMP-8. The *x* axis indicates which CSTs were compared, and the arithmetic relationship was plotted. The central point is the estimate, and the bars indicate the 95% confidence interval on the difference; significance is given by the color of the bar and calculated using a Tukey test. Download FIG S3, DOCX file, 0.1 MB. Copyright © 2019 Witkin et al. 2019 Witkin et al. This content is distributed under the terms of the Creative Commons Attribution 4.0 International license.

10.1128/mBio.02242-19.6 FIG S4 Boxplots of immunological data broken down by CST. Each panel shows the distribution of the normalized (by total protein content) and transformed (by hyperbolic arcsine) raw data for each variable according to bacterial CST of the vagina. Download FIG S4, DOCX file, 0.1 MB. Copyright © 2019 Witkin et al. 2019 Witkin et al. This content is distributed under the terms of the Creative Commons Attribution 4.0 International license.

### Prediction of cervical length.

Stepwise regression of cervix length on potential explanatory variables yielded a fairly simple model. The final model (Table 3) included d-lactic acid, TIMP-1, p62, age, and race as predictors. Of those predictors, cervix length was positively associated with only d-lactic acid and age. Stepwise regression improved the Akaike information criterion (AIC) of the model from 2,391 to 2,379, while reducing the predictive power only slightly (*R*^2^ dropped from 0.158 to 0.143).

**TABLE 3:** Results of linear model selection to predict cervical length

Independent variable	Value for variable (95% CI) in predicting cervical length
d-Lactic acid	131.94 (−39.35, 303.23)
TIMP-1	−0.89 (−1.49, −0.30)^*b*^
p62	−70.35 (−146.38, 5.68)^*a*^
Age	0.23 (0.11, 035)^*b*^
Mixed race	0.36 (−1.46, 2.17)
Black race	−5.08 (−8.01, −2.16)^*b*^
Constant	28.27 (23.95, 32.60)^*b*^
No. of observations	340
*R*^2^	0.143
Adjusted *R*^2^	0.128
Residual SE	7.89 (df = 333)
*F* statistic	9.30 (df = 6; 333)^*c*^

a Numbers given for the slope estimates are the estimate with the 95% confidence interval (CI) in parentheses.

b *P* < 0.01.

c *P* < 0.1.

**FIG 4:**
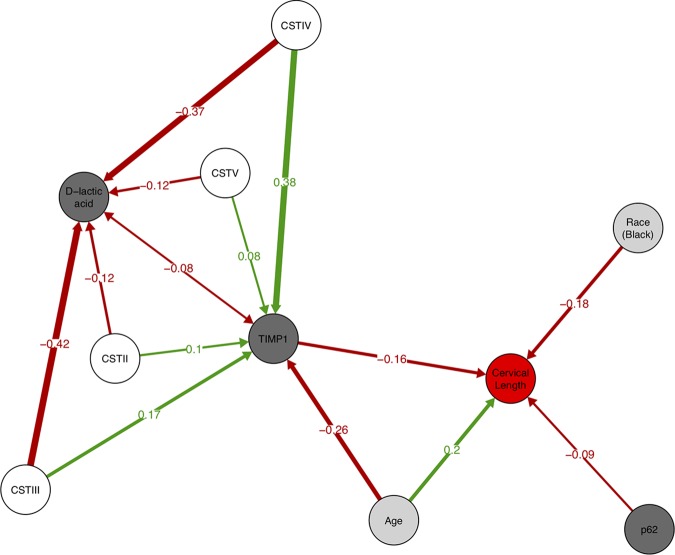
Path diagram based on significant relationships (*P* < 0.1) detected by piecewise SEM. Red arrows indicate negative relationships, and green arrows indicate positive relationships. Path coefficients are the standardized regression coefficients and show the relative strength of effect for particular relationships. Single-headed arrows indicate direct relationships between two variables; the double-headed arrow between d-lactic acid and TIMP-1 indicates an unexplained correlation. The strength of the effect of variable on cervix length is the sum of the direct and indirect effects. For example, age has a direct effect of 0.2 and an indirect effect via TIMP-1 of −0.26 × −0.16; thus, the net effect of age is 0.2 + –0.26 × –0.16 = 0.2416. Likewise, CST IV has only indirect effects which give a net effect of –0.37 × –0.08 × –0.16 + 0.38 × –0.16 = –0.0655. Circles are color coded according to the type of variable (microbiome, immunological, host identity, and cervix length).

Combining the cervical length model with the models for d-lactic acid and TIMP-1 using piecewise structural equation modeling (SEM) allowed us to estimate direct and indirect effects of predictors on cervix length. When we first combined the three models, tests of directed separation suggested that several important features were missing from our path model (*C* = 243.0, *df* = 16, *P* < 0.001). Specifically, there were missing relationships between the three immunological variables (TIMP-1 with d-lactic acid, TIMP-1 with p62, and d-lactic acid with p62) as well as effects of age and race on TIMP-1 and d-lactic acid. All of these paths were subsequently added to the model. Relationships between vaginal compounds were treated as unexplained correlations, while age and race were postulated to have direct effects on TIMP-1 and d-lactic acid. After addition of these paths, the model fit was greatly improved (*C* = 5.3, *df* = 2, *P* = 0.072). The AIC comparison of the two models yielded values of 283.0 (initial model) and 57.3 (improved model), thus strongly favoring the latter (Table 4). Interestingly, there was marginal evidence (*P* = 0.072) of the CST having a direct effect on cervix length. A path diagram derived from the piecewise SEM analysis (Fig. 4) illustrates where direct and indirect effects play a role in predicting cervix length in pregnant women.

**TABLE 4:** Paths suggested by the SEM modeling process

Response^*a*^	Predictor^*b*^	Estimate^*c*^	SE^*c*^	Std estimate^*d*^	*P* value^*e*^
Cervical length	d-Lactic acid	131.94	87.39	0.0786	0.1321
	TIMP-1	−0.89	0.30	−0.1616	0.0035**
	p62	−70.35	38.70	−0.0938	0.0706!
	Age	0.23	0.06	0.1961	0.0003***
	Mixed race	0.36	0.93	0.0205	0.6990
	Black race	−5.08	1.49	−0.1806	0.0007***

TIMP-1	CST II	0.96	0.50	0.0953	0.0549!
	CST III	0.57	0.17	0.1737	0.0012**
	CST IV	1.53	0.21	0.3826	0.0000***
	CST V	0.58	0.35	0.0830	0.0982!
	Age	−0.05	0.01	−0.2583	0.0000***
	Mixed race	−0.12	0.16	−0.0371	0.4645
	Black race	0.38	0.26	0.0735	0.1486

d-Lactic acid	CST II	−0.004	0.002	−0.1242	0.0135*
	CST III	−0.005	0.001	−0.4183	0.0000***
	CST IV	−0.005	0.001	−0.3866	0.0000***
	CST V	−0.003	0.001	−0.1170	0.0214*
	Age	0.000	0.000	0.0288	0.5647
	Mixed race	−0.0004	0.001	−0.0409	0.4362
	Black race	−0.0002	0.001	−0.0133	0.7956

TIMP-1	∼∼d-lactic acid	−0.08	−0.0830	0.0636	
	∼∼p62	0.05	0.0515	0.1729	

d-Lactic acid	∼∼p62	−0.02	−0.0245	0.3267	

a The response column shows the variable affected by the corresponding entry in the predictor columns.

b Entries marked with two tildes indicate bidirectional unexplained correlations between variables.

c The estimate and its standard error (SE) come from the component linear regression models.

d The standardized estimate (Std estimate) is given to show the relative effect (path) strengths.

e The *P* values are given. Symbols: !, *P* < 0.1; *, *P* < 0.05; **, *P* < 0.01; ***, *P* < 0.001.

## DISCUSSION

Preterm birth is a major public health concern due to associated morbidity and mortality, particularly in resource-poor areas where the means to apply appropriate analytical tools to measure risk factors and initiate preventative measures are often lacking. To aid in the development of inexpensive point-of-care diagnostics for two key factors associated with preterm birth risk, we modeled the relationship between the vaginal microbiome, compounds in vaginal secretions, and cervical length in a large cohort of women from Sao Paolo, Brazil. We identified factors that influence cervical length in pregnant women. Specifically, TIMP-1, d-lactic acid, p62, age, and race all directly affected cervical length. Additionally, there was weak evidence (*P* = 0.072) that the microbiome composition may also have a direct effect. An association between cervical length and the composition of the vaginal microbiome in women analyzed at 16 weeks gestation has been previously reported (8). In this study, we observed that the microbiome had an indirect effect on cervical length via their influence on TIMP-1 and d-lactic acid concentrations. We also observed an indirect association between maternal age and decreasing levels of TIMP-1 and a significant, but a weak negative correlation between TIMP-1 and d-lactic acid (*r*  = -0.08).

TIMP-1, p62, and belonging to the black race had strong negative effects on cervical length (standardized regression coefficients of –0.162, –0.094, and –0.181, respectively). Elevated TIMP-1 levels have been previously associated with earlier gestational age at the time of delivery (17, 24, 25). Additionally, it was reported that TIMP-1 is suppressed during normal pregnancy with levels increasing periparturition (17). In contrast to prior studies on smaller numbers of subjects (17, 26), we did not find an association between either MMP-2 or MMP-8 and cervical length, and there was no evidence of MMPs playing a significant role beyond that which could be explained by TIMP-1 alone. p62 is a protein that is degraded by lysosomal enzymes during the activation of autophagy. Its intracellular concentration, therefore, is inversely correlated to the extent of autophagy (27). A function of autophagy is to identify and kill intracellular pathogens. Thus, this process is upregulated during infection and proinflammatory states (28). These conditions increase susceptibility to preterm birth (28).

Sociodemographic variables are known to influence preterm birth (29–31). In our study, the influence of black race on cervical length was as strong as being either 1 standard deviation above the mean (84th percentile) in TIMP-1 levels, or 2 standard deviations above the mean in p62 concentration (98th percentile). Some studies have suggested that the risk of preterm birth may be up to twice as high in black women compared to nonblack women (29). Likewise, age is a known risk factor for preterm birth (32, 33). Consistent with our study are observations that younger women are more likely to experience preterm birth. Interestingly, we found that age has an indirect positive effect on reducing preterm birth via a reduction of TIMP-1 concentrations. Thus, the net effect of age on cervical length is quite strong (direct effect = 0.196, indirect effect = –0.162 + –0.258 = –0.042, net effect = 0.042 + 0.196 = 0.238). However, a previous study on age and preterm birth showed that at older ages (>40 years), the risk of preterm birth rises (33). We may not have observed this effect because of the limited age range of our study population.

With regard to the microbiome, we observed several interesting patterns. All of the significant effects of the CST groups on cervical length were via indirect effects on TIMP-1. CST IV, often associated with high levels of *G. vaginalis*, had the highest levels of TIMP-1 and the strongest indirect effect on cervical length. This finding fits with previous results which have shown that bacterial vaginosis, which is associated with CST IV, is a risk factor for preterm birth (13). Communities dominated by *L. iners* (CST III) also exhibited increased levels of TIMP-1 and negative indirect effects on cervical length. This observation is also consistent with prior evidence that *L. iners* is associated with adverse pregnancy outcomes (6, 34). Vaginal communities dominated by L. crispatus (CST I) had the lowest levels of TIMP-1, followed closely by communities dominated by L. gasseri and L. jenseni. This observation is consistent with studies showing that vaginal dominance by these *Lactobacillus* species promotes healthy pregnancy progression (5–10, 34, 35).

There were also significant effects of CST on d-lactic acid concentrations, but the effect of d-lactic acid on cervical length, while positive, was not significant. The pattern of influence on d-lactic acid was almost diametrically opposed to that of TIMP-1. CST I was clearly associated with the highest level of d-lactic acid, followed by CST V. This fits with prior findings that L. crispatus and L. jensenii are known to produce d-lactic acid (16). Conversely, CST II and IV were associated with the lowest levels of d-lactic acid. By combining data regarding microbiome associations with TIMP-1, we hypothesize that CST I, which produces high levels of d-lactic acid and induces low levels of TIMP-1, should have the lowest risk of preterm births. Conversely, CST IV communities should have the highest risk of preterm birth. We also hypothesize that CST V is similar to CST I with regard to preterm birth risk, while CST III is similar to CST IV.

Humans are the only mammal in which the vaginal microbiota of most reproductive age females are dominated by lactobacilli (36, 37). The abundance of lactobacilli in the vagina increases further during pregnancy (38, 39). It has been proposed that this distinctive vaginal microbiome evolved to best preserve fecundity in response to the unique human lifestyle and environmental exposures (37). The association of the vaginal d-lactic acid concentration with dominance of the vaginal microbiota by distinct species of lactobacilli species in the present study further supports a role for this isomer in promoting vaginal health. In addition to the production of d-lactic acid, L. crispatus may also contribute to pregnancy well-being by preventing the proliferation of other bacteria that have been associated with increased susceptibility to adverse pregnancy outcome.

A limitation of the study was our inability to relate lactic acid and TIMP-1 levels or microbiome composition with pregnancy outcome. As mentioned in Materials and Methods, women with a cervical length of <25 mm received prophylactic vaginal progesterone treatment. As a consequence, we identified risk factors in a population that probably had a reduced overall risk. This may explain why the amount of variance in cervix length explained by our simple model was relatively low (*R*^2^ = 0.143). In addition, cervical length may also be modified by factors unrelated to the vaginal microbiota. This likely also influenced the extent of the observed associations. We did not have information on the incidence of preterm premature rupture of membranes (pPROM) in our subjects. An association between pPROM and the vaginal microbiota has been previously reported (40). A further limitation was our inability to collect clinical and pregnancy-related data from all of the subjects in which microbiome analysis and lactic acid levels were determined. However, all data from subjects with known outcomes did not differ significantly from those with missing clinical data. Thus, it is unlikely that additional data would have significantly altered our findings or interpretation of the data. It should be mentioned that the Brazilian population has a unique admixture of races (41), and our findings need to be confirmed in other populations of pregnant women. Also, our evaluation of compounds in vaginal secretions was selective, and a more exhaustive evaluation may find other entities that also influence cervical length. In fact, a recent study concluded that the vaginal concentration of beta-defensin-2 influenced pregnancy outcome, even when controlling for the abundance of lactobacilli (10). Last, our study aimed to evaluate associations between cervical length, composition of the vaginal microbiome, and compounds in vaginal secretions. Due to the limitations mentioned above, the study was not intended to assess the diagnostic value of our findings for pregnancy outcomes in our subject population. Instead, our observations provide a first step toward the development and use of inexpensive point-of-care diagnostic tests to assess the presence of known risk factors for preterm birth in resource-poor areas. The measurement of TIMP-1, d-lactic acid, and p62 concentrations appears to provide reasonable predictive power for risk assessment based on short cervical length. Furthermore, measurements of TIMP-1 also provide an indirect assessment of the dominant bacteria present in the vaginal microbiome during pregnancy without the need for either microscopy or gene sequencing. It would be of major interest to replicate the present study in first trimester pregnant women to ascertain whether measurement of vaginal d-lactic acid and/or TIMP-1 at this stage of gestation and treatment of women at risk will also reduce the incidence of preterm birth in disadvantaged populations. Finally, our results suggest that subsequent development of protocols to drive the vaginal microbiome toward CST I may have cascading health benefits for the prevention of preterm birth via indirect effects on both d-lactic acid and TIMP-1.

## MATERIALS AND METHODS

### Clinical study.

**(i) Subjects.** The participants (see Table S1 in the supplemental material) in this prospective study were mid-trimester pregnant women who were undergoing a routine vaginal ultrasound to assess cervical length at the obstetrical outpatient clinic at The Federal University of Sao Paulo. Patients were a mixture of women at low risk for a preterm birth and those with an identified characteristic that placed them at elevated risk: short cervical length, history of preterm birth or spontaneous miscarriage, vaginal bleeding in the first trimester, or obesity. For ethical reasons, women with a short cervical length (<25 mm) received prophylactic treatment consisting of a 200-mg dose daily of vaginal progesterone (Utrogestan) until 36 weeks gestation or delivery. Cervical cerclage was not used. Exclusion criteria were the presence of a multifetal gestation, signs or symptoms suggestive of a vaginal infection, antibiotic usage in the previous 2 weeks, presence of an immune or endocrine disorder, or the inability to give informed consent. The study was approved by the Institutional Review Board at The Federal University of Sao Paulo, and all subjects gave written informed consent.

**(ii) Samples.** Just prior to the cervical length assessment, samples were obtained from the posterior vagina for the analysis of vaginal compounds and the composition of the vaginal microbiome. For the vaginal compound determination, samples obtained with a cotton swab were vigorously shaken into a sterile tube containing 1 ml of sterile phosphate-buffered saline. The tube was centrifuged, and the supernatant was stored in aliquots at –80°C. The epithelial cell pellet was immediately lysed with a detergent-protease inhibitor-containing buffer as previously described (42) and centrifuged, and the lysate was stored at –80°C. For the microbiome analysis, samples were collected using the Copan ESwab sample collection system (Fisher Scientific, Pittsburgh, PA) and stored at –80°C. All samples were placed in dry ice and shipped to the Witkin lab at Weill Cornell Medicine, and microbiome samples were subsequently shipped on dry ice to the Forney lab at the University of Idaho. All lab assays were performed by staff blind to all clinical information.

**(iii) Vaginal compound measurements.** Vaginal levels of the d- and l-lactic acid isomers were quantitated by colorimetric assays using the EnzyChrom d-lactic acid and l-lactic acid kits (BioAssay Systems, Haywood, CA). The levels of TIMP-1, TIMP-2, MMP-2, MMP-8, and Hsp70 (all from R&D Systems, Minneapolis, MN) and total protein (Thermo-Fisher Scientific, Waltham, MA) in the vaginal fluid supernatant and p62 (Enzo Life Sciences, Farmingdale, NY), the a2 isoform of vacuolar ATPase (a2V) (My BioSource, San Diego, CA), and total protein in the lysed epithelial cell fraction were determined by commercial enzyme-linked immunosorbent assay (ELISA) kits. Values were first converted to picograms per milliliter or millimolar by reference to a standard curve that was generated with each assay and then to picograms, nanograms, or micromolar per microgram of total protein in each individual sample.

**(iv) Cervical length measurement.** Cervical length evaluation by transvaginal sonography was performed with a 5- to 9-MHz probe (Accuvix XQ and V10, Medison, South Korea; Voluson Expert 730, USA) according to standard techniques. Women were asked to empty their bladders and then placed in the dorsal lithotomy position, and the transducer was inserted in the anterior vaginal fornix. The cervix was visualized in the longitudinal plane, the endocervical mucosa was identified, and the cervical length was measured as the distance from the internal os to the external os. The shortest cervical length measurement was recorded after 3 to 5 min of transvaginal sonography. Transfundal pressure was applied in order to note adverse dynamics and funneling of the cervix. The ultrasound examinations were performed by experienced physicians with fetal medicine training background.

### Vaginal microbiome.

**(i) Sequences of 16S rRNA genes.** The microbiome analyses were performed as previously reported (42). Briefly, bacterial cells in vaginal samples were lysed using an enzyme cocktail and bead beating, and genomic DNA was isolated using a QIAamp DNA minikit. DNA yield was determined by fluorometry, and the DNA size and integrity were verified using an Agilent Bioanalyzer. The V1 to V3 regions of bacterial 16S rRNA genes were amplified using primers that flanked the variable regions, and amplicons were produced by two consecutive rounds of PCR that attached sample barcodes and sequencing adapters. The concentrations of amplicons were determined by fluorometry. DADA2 software (v1.8) was used to identify distinct sequence variants (DSVs) and remove sequence chimeras. These DSVs were classified to the genus level using the RDP naive Bayesian classifier (v11.5) in combination with the SILVA reference database and then assigned to species using SPINGO software. Data were cleaned to include only samples with more than 3,000 reads.

**(ii) Microbiome simplification.** After initial determination of the DSVs in the vaginal microbiomes, 222 different species were identified in the 629 samples analyzed (Table S2). This number of species was too large to perform statistical analyses, and thus, simplification of the microbiome data was necessary. To do this, we filtered the data in three ways in which each filter was based on a measure of dominance in the community. For a species to be retained for analysis, it had to be in the top 15 of: (i) mean rank abundance, (ii) mean relative abundance, and (iii) total read counts. Species that did not meet these three criteria were placed into a new category simply called “other”; thus, no sequencing reads were lost in the simplification process. After applying these filters, the 222 species were reduced to 10 species: Lactobacillus crispatus, *L. iners*, L. gasseri, L. jensenii, Gardnerella vaginalis, Atopobium vaginae, *Megasphaera* spp., Aerococcus christensenii, *Prevotella* spp., and other (the sum of all read counts not in the previous 9 species).

### Statistics.

**(i) Sample sizes.** Both microbiome data and vaginal compound data were collected from enrollees, as well as cervical length and other metadata. Of the 629 women for whom we have microbiome data, 420 also had recorded cervical length measurements. Within this set of 420 women, we selected for analysis those subjects in which the measurement of compounds in the vagina was relatively complete, i.e., there were less than 20 missing measures and the associated microbiome and cervix length measurements were available. This resulted in a sample size of 340 for the statistical analyses that involved vaginal compound measurements. The compounds measured included TIMP-1, TIMP-2, d-lactic acid, l-lactic acid, MMP-2, MMP-8, a2V (vaginal lysate), Hsp70 (vaginal lysate), p62 (vaginal lysate), total protein, and total protein in the vaginal lysate.

**(ii) Characterization of the vaginal microbiome.** We performed three types of analysis on the simplified microbiome data. First, clustering analysis was performed. Second, plotting of the communities in a two-dimensional (2D) space was accomplished using nonmetric dimensional scaling (NMDS). Third, we characterized the overall mean of the relative abundances within the samples, as well as the means for the clusters revealed by the first analysis. All analyses herein were performed using R v 3.5 (43).

To perform the first two analyses, a distance metric was used to describe how close communities were to each other in multidimensional space (in this case, 10 dimensions). We chose to use the measure proposed by Anderson et al. (44) (see equation 6 therein), which measures the mean absolute difference between all nonzero pairwise observations (i.e., if L. crispatus were 0 in the two communities being compared, it would be excluded from the mean calculation, which fixes the problem of zero pairs being overweighted in distance calculations). For both clustering and NMDS, identical communities were removed from the sample, bringing the sample size to 428 unique communities and 201 duplicated communities.

Clustering of the unique communities was performed by the hclust algorithm using the unweighted-pair group method with arithmetic means (UPGMA) (22). To determine the number of “significant” clusters, we applied the silhouette method (23). which chooses the number of clusters based on the maximum silhouette width. The basic concept of the silhouette is to examine how the average relatedness within clusters covaries (changes) with the number of clusters. Clusters were subsequently labeled according to the appropriate vaginal community state type (CST).

Once the appropriate number of clusters of was determined, the metaMDS algorithm (in the vegan package) (45), was used to perform NMDS to reduce the dimensionality of the data to the same number. We ensured that the stress measurement (the percentage of information lost during reduction) was sufficiently low. We used the first two axes (those that explain the greatest differences between communities) to plot the unique communities in the NMDS transformed space.

**(iii) Analysis of vaginal fluid data.** We performed a simple transformation of the vaginal compound data prior to analysis. First, we standardized the respective measures by the total protein content in the sample from each subject. Second, the distributions of most of the standardized data were exponential and thus did not meet the statistical assumptions of the typical linear model (namely, independent and identically distributed residuals [i.i.d. residuals]). To correct for this issue, we performed a hyperbolic arcsine transform that is similar to a log transform but is less sensitive to zeroes in the data. Standardization and transformation greatly improved the distribution of the residuals from our analyses.

We determined whether specific CSTs were associated with concentrations of the individual compounds in the vaginal samples. Simple linear models with a marker (e.g., TIMP-1, TIMP-2) as the dependent variable and bacterial community types as the independent variable were constructed. If the linear model was significant, we then performed Tukey’s test to identify differences between community means. For example, if the model for TIMP-1 versus CST was significant, we then performed all pairwise comparisons of group means of TIMP-1 (i.e., comparing the means of the individual five CSTs against each other [CST I versus CST II, I versus III, I versus IV, …]), the *P* values were adjusted to maintain a 5% family-wise error rate.

**(iv) Analysis of cervix length.** We combined the cervical length data with our vaginal compounds, microbiome, and other metadata to create a predictive model of cervical length. The other metadata used included the subject’s age, race (white, black, or mixed), and body mass index (BMI). We wanted to create a simple, parsimonious predictive model. Therefore, we used a stepwise linear regression procedure that started with the inclusion of all vaginal variables, age, race, and body mass index. The Akaike information criterion (AIC) was used to determine which variables to drop from the model or add to the model (both forward and backward variable selection was used).

**(v) Direct and indirect effects on cervix length.** To look for potential causal pathways that include direct and indirect effects, we used piecewise structural equation modeling (SEM) (46). Piecewise SEM takes component models and places them together in a path analysis. For example, component model A might be Y ∼ X, and component model B might be Z ∼ Y; the SEM model would then be Z ∼ Y ∼ X, which suggests X indirectly affects Z via Y but has no direct effect itself on Z (i.e., Z ∼ X). We used the piecewise SEM package (43) to synthesize our cervix length model with our vaginal secretion models to look for direct and indirect effects of the microbiome CST, vaginal compound status, and host identity on cervix length. Specifically, the vaginal compound models included in the path analysis were those for d-lactic acid and TIMP-1, because we found significant associations between bacterial community cell type and these variables (see Results). Using tests of directed separation and AIC (46), we either added or removed paths between variables in the analysis. Path coefficients (standardized regression coefficients) were calculated *post hoc* to compare the relative strength of effects within the path network.
