# Supplementary material for: Vaginal Biomarkers That Predict Cervical Length and Dominant Bacteria in the Vaginal Microbiomes of Pregnant Women
Source: mBio. 2019 Oct 22;10(5):e02242-19. doi: 10.1128/mBio.02242-19 (PMC6805993; doi:10.1128/mBio.02242-19)
Supplement: TABLE S1 [file mBio.02242-19-st001.docx]

Supplemental Table 1. Demographic data on 629 women used for microbiome analysis.

_________________________________________________________

Characteristic Mean SD^a^ Median

_________________________________________________________

Age (years) 29.2 7.4 29.0

Body mass index (km/m2) 27.4 6.2 26.4

Race

White 53.9%

Mixed 36.5%

Black 9.6%

Cervical length (mM) 32.4 9.0 33.2

Short cervix (≤25 mM) 12.5%

Gravidity 2.5 1.6 2.0

Parity 1.0 1.2 1.0

Gest. age sample (weeks) 21.4 1.4 21.4

Gest. age delivery (weeks)^a^ 38.3 2.6 38.9

Preterm birth (<37 weeks)^a^ 15.4%

________________________________________________________

^a^ SD = standard deviation
