# Supplementary figures and images for: Vaginal Biomarkers That Predict Cervical Length and Dominant Bacteria in the Vaginal Microbiomes of Pregnant Women
Source: mBio. 2019 Oct 22;10(5):e02242-19. doi: 10.1128/mBio.02242-19 (PMC6805993; doi:10.1128/mBio.02242-19)

**Supplemental Figure S1**


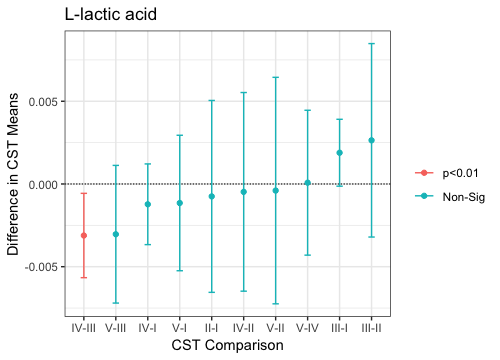

Supplement: FIG S1 [file mBio.02242-19-sf001.docx]

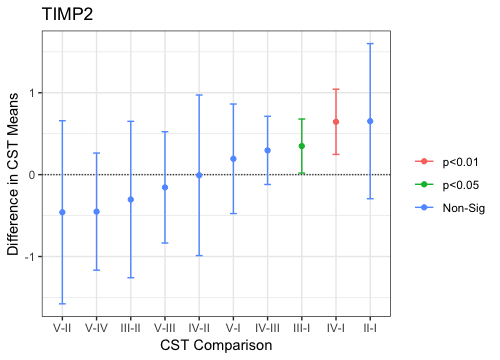
**Supplemental Figure S2**

Supplement: FIG S2 [file mBio.02242-19-sf002.docx]

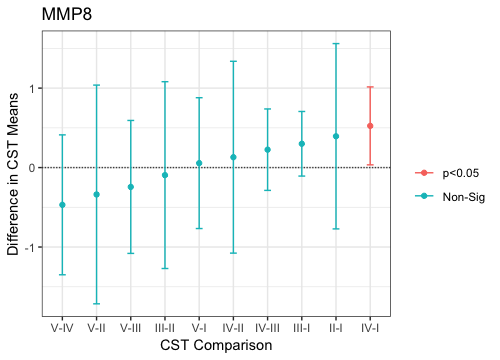
**Supplemental Figure S3**

Supplement: FIG S3 [file mBio.02242-19-sf003.docx]
