# Supplementary material for: Vaginal Biomarkers That Predict Cervical Length and Dominant Bacteria in the Vaginal Microbiomes of Pregnant Women
Source: mBio. 2019 Oct 22;10(5):e02242-19. doi: 10.1128/mBio.02242-19 (PMC6805993; doi:10.1128/mBio.02242-19)
Supplement: FIG S4 [file mBio.02242-19-sf004.docx]

Supplemental Figure S4
